# The leukemia-associated RUNX1/ETO oncoprotein confers a mutator phenotype

**DOI:** 10.1038/leu.2015.133

**Published:** 2015-06-30

**Authors:** V J Forster, M H Nahari, N Martinez-Soria, A K Bradburn, A Ptasinska, S A Assi, S E Fordham, H McNeil, C Bonifer, O Heidenreich, J M Allan

**Affiliations:** 1 Northern Institute for Cancer Research, Newcastle Cancer Centre, Newcastle University, Newcastle-upon-Tyne, UK; 2 School of Cancer Sciences, Institute of Biomedical Research, College of Medical and Dental Sciences, University of Birmingham, Birmingham, UK

t(8;21) is a frequent chromosomal translocation in acute myeloid leukemia (AML) and is also reported in lymphoid and biphenotypic acute leukemia.^1, 2^ t(8;21) fuses the *RUNX1* gene (*AML1*) on chromosome 21 to the *ETO* gene (*RUNX1T1*) on chromosome 8, encoding the RUNX1/ETO chimeric transcription factor that represses expression of RUNX1 target genes, promoting self-renewal and blocking myeloid differentiation.^3, 4, 5, 6^ t(8;21) is insufficient for leukemogenesis and additional co-operating mutations are required for transformation,^7^ including point mutations that activate and/or over express c-KIT.^8^ The mechanisms driving the acquisition of co-operating mutations remain unclear, although there is evidence that initiating lesions such as RUNX1/ETO may promote mutagenesis.^9, 10^ For example, ectopic expression of RUNX1/ETO downregulates several DNA-repair proteins (BRCA2, OGG1 and ATM) and increases the level of phosphorylated TP53 and γH2AX, indicating elevated DNA damage and a possible pro-mutagenic phenotype.^10, 11^

To test whether expression of RUNX1/ETO is sufficient to increase genomic instability and mutagenesis, we expressed RUNX1/ETO in the non-transformed TK6 lymphoblastoid cell line and measured the acquisition of mutations at the *PIGA* reporter gene. Cells were transduced with lentivirus carrying either enhanced green fluorescent protein (*EGFP)* and the *RUNX1/ETO* fusion or *EGFP* alone (vector control) (Supplementary Figure 1A; Supplementary Methods) and clones expressing both RUNX1/ETO mRNA and protein were generated (Supplementary Figures 1B and C). For the purposes of measuring RUNX1/ETO-induced mutagenesis, we analysed independent cell clones expressing at least 40% of the fusion transcript found in the Kasumi-1 and SKNO-1 t(8;21)-positive cell lines (Supplementary Figure 1B). Monitoring of RUNX1/ETO-positive clones revealed that expression of the fusion was stable over long culture periods (>12 weeks). Independent vector control clones that expressed equivalent levels of EGFP to RUNX1/ETO-positive cells were used as fusion-protein negative controls (Supplementary Figure 1D).

*PIGA* encodes a protein essential for production of the glycosylphosphatidylinositol anchor, mediating anchoring of proteins including CD55 and CD59 to the cell membrane.^12^ Somatic mutations of *PIGA* are growth neutral and can be determined using flow cytometry analysis measuring CD55 and CD59 expression (Supplementary Figure 2; Supplementary Methods). Using this reporter gene system we determined the mutation frequency (M*f*) in several independent RUNX1/ETO-expressing and vector control clones after continuous culture for 8–10 weeks after initial cloning. All clones had a majority population that was positive for CD55 and CD59, confirming that the founding cell was PIGA wild-type (WT). The mean *PIGA* M*f* in RUNX1/ETO clones (7.50 × 10^−4^) was five times higher than in vector control clones (1.45 × 10^−4^) (Figure 1a, Supplementary Figure 3A) (*P*=0.032), with the exception of a single clone that had low expression of the fusion transcript (equivalent to 40% of that in Kasumi-1) (Supplementary Figures 1B and 3A).

The frequency of cells with a mutation (M*f*) at a specific gene is determined by the mutation rate per cell division for that gene (*μ*) and the number of cell divisions (*d*). Expression of the RUNX1/ETO fusion had a modest but nonsignificant negative effect on cell proliferation (Figure 1b, Supplementary Figure 3B, *P*=0.812), confirming that the increased M*f* in RUNX1/ETO fusion-positive cells was not due to elevated proliferation.

Despite being derived from single cells, EGFP expression in RUNX1/ETO clones was normally distributed in all cell populations (Figure 1c, Supplementary Figure 4), indicating a natural drift in EGFP post cloning. Our data suggest that RUNX1/ETO confers a mutator phenotype in a protein-level-dependent fashion. By using EGFP as a surrogate for RUNX1/ETO we predicted that *PIGA-*mutant cells would have higher EGFP expression compared with non-mutant cells from the same population. Consistent with this hypothesis, the EGFP geometric mean of fluorescence (GMoF) of *PIGA* mutant cells derived from RUNX1/ETO cell clones was significantly higher than in non-mutants derived from the same starting population (Figures 1c and d, Supplementary Figure 4, Supplementary Table 1). The only RUNX1/ETO clone that did not show a difference between EGFP GMoF for *PIGA* WT and mutant cells was that with the lowest RUNX1/ETO expression (clone RE4), which also had no increase in spontaneous *PIGA* M*f* (Supplementary Figure 3A). For vector control clones, the EGFP GMoF was not significantly different between *PIGA* mutant and WT cells (Figure 1d, Supplementary Figure 4, Supplementary Table 1). These data demonstrate that expression of the RUNX1/ETO fusion oncoprotein confers a mutator phenotype when the transcript is at levels equivalent to at least 60% of that in t(8;21)-positive cell lines.

We next investigated whether RUNX1/ETO transduced cells were particularly sensitive to mutagenesis following exposure to genotoxic anticancer therapies. Fusion protein-positive and vector control clones were exposed to sub-cytotoxic doses of either doxorubicin (100 nM for 4 h) or ionising radiation (3 Gy), and M*f* was measured 2 weeks after treatment. The frequency of mutations attributable to doxorubicin or radiation (treatment-induced M*f*) was calculated by subtracting the M*f* in mock-treated cells from the M*f* in doxorubicin- or radiation-treated cells. The mean treatment-induced *PIGA* M*f* was strongly inceased in RUNX1/ETO cell clones compared with vector control clones following either doxorubicin (mean M*f*=6.54 × 10^−4^ vs 0.208 × 10^−4^, *P*=0.09) or radiation treatment (mean M*f*=5.92 × 10^−4^ vs 0.209 × 10^−4^, *P*=0.008) (Figure 2a, Supplementary Figure 5). As an additional independent measure for treatment-induced M*f* we assayed RUNX1/ETO and vector control clones at a second reporter gene, thymidine kinase (*TK*). The mean treatment-induced *TK* M*f* was also significantly higher in RUNX1/ETO cell clones compared with vector control clones following either doxorubicin (mean M*f*=3.53 × 10^−6^ vs 0.432 × 10^−6^, *P*=0.014) or radiation treatment (mean M*f*=6.43 × 10^−6^ vs −0.145 × 10^−6^, *P*=0.002) (Figure 2a, Supplementary Figure 5). The only RUNX1/ETO clone that did not show increased sensitivity to doxorubicin or radiation-induced mutation was clone RE4 with low RUNX1/ETO expression (Supplementary Figure 5). For both *PIGA* and *TK*, the M*f* in vector control clones following doxorubicin or radiation treatment was not significantly different from mock-treated cells (Figure 2a, Supplementary Figure 5), suggesting that, unlike RUNX1/ETO-expressing cells, vector control cells were proficient at repairing treatment-induced DNA damage.

We next investigated whether EGFP expression differed in treatment-induced *PIGA* mutant cells compared with non-mutant cells derived from the same RUNX1/ETO clonal population. The EGFP GMoF of *PIGA* mutant cells was significantly higher than in non-mutant cells following treatment with doxorubicin (*P*=0.03) or ionising radiation (*P*=0.02) (Figures 2b and c, Supplementary Figure 6). In contrast, the EGFP GMoF was not significantly different between *PIGA* mutant and WT cells derived from vector control populations following treatment (Figures 2b and c).

These data demonstrate that high levels of RUNX1/ETO sensitise cells to acquisition of mutations following treatment with DNA damage-inducing agents, suggesting that fusion-protein-expressing cells may be compromised in DNA repair. This result is consistent with previously published reports implicating compromised OGG1 DNA glycosylase activity in t(8;21) AML.^11, 13^ OGG1 functions in base excision repair and initiates repair of oxidised lesions, including 8-hydroxy-2′-deoxyguanosine. We therefore investigated *OGG1* expression in 18 independent RUNX1/ETO cell clones and observed that transcript levels were inversely proportional to the expression of the fusion gene (Supplementary Figure 7A). Likewise, OGG1 protein levels were consistently lower in RUNX1/ETO-positive clones compared with vector control clones (Supplementary Figure 7B). We next performed siRNA-mediated depletion of *RUNX1/ETO* in Kasumi-1, as previously described.^5^ Two serial electroporations with either *RUNX1/ETO* or control scrambled siRNA were carried out and *OGG1* transcript and protein levels were assessed 72 h after each electroporation. RUNX1/ETO depletion increased *OGG1* transcript levels by fourfold and sixfold on days 3 and 6, respectively (Supplementary Figure 7C) with a concomitant increase in OGG1 protein (Supplementary Figures 7C and D). To confirm *OGG1* as a RUNX1/ETO target gene we performed ChIP and RNA sequencing after siRNA-mediated *RUNX1/ETO* depletion. A RUNX1/ETO CHiPseq peak was observed at the *OGG1* promoter in control-transfected Kasumi-1 cells, which was lost following siRNA-mediated fusion protein depletion (Supplementary Figure 7E) and was co-localised within a DNaseI hypersensitivity site, as previously reported.^6^ We also confirmed the increase in *OGG1* expression after RUNX1/ETO depletion by RNAseq (Supplementary Figure 7E). Taken together, these data confirm that the RUNX1/ETO fusion protein binds the *OGG1* promoter and negatively regulates transcription, suggesting a plausible mechanism by which this common fusion oncoprotein drives mutagenesis. In order to investigate this further, we interrogated whole-genome and exome sequencing data from The Cancer Genome Atlas Project.^14^ Data were available from 193 AML cases, which included information on 21 386 somatic base substitution mutations, including G>T transversions, the predominant mutation that arises following translesion synthesis of 8-hydroxy-2′-deoxyguanosine and which accumulates following knockdown of OGG1 in experimental systems.^15^ When stratified by cytogenetic subgroup, 16.13% of all mutations in t(8;21) AML were G>T transversions (16.13%, *n*=7 cases, Supplementary Table 2), which was higher than any other cytogenetic subgroup and all other AML cases combined (12.14%, *n*=186 cases), although this did not reach statistical significance (*P*=0.31, Yates chi-square). These data are consistent with loss of OGG1 contributing to the mutator phenotype in RUNX1/ETO cells by predisposing to G:C>T:A transversions.

By using mutation assays using two independent assays, we have shown that RUNX1/ETO predisposes to the acquisition of mutations, both spontaneously and particularly after treatment with genotoxic agents. We also present evidence that the strength of the mutator phenotype is related to the expression level of the fusion protein. As with all types of AML, relapse is a major cause of mortality in t(8;21) AML. If remission–induction chemotherapy fails to eliminate all the RUNX1/ETO-positive cells, our data suggest that surviving cells are predisposed to mutations that could drive relapse. One consideration for chemotherapy should therefore be to limit mutation of surviving leukemic/preleukemic cells. As such, we hypothesise that targeting the mutator phenotype associated with expression of the RUNX1/ETO fusion gene could impede the acquisition of co-operating mutations required for disease relapse. Our data demonstrate the need to develop novel therapeutic strategies that avoid increasing the mutation burden of AML cells.

## Figures and Tables

**Figure 1:**
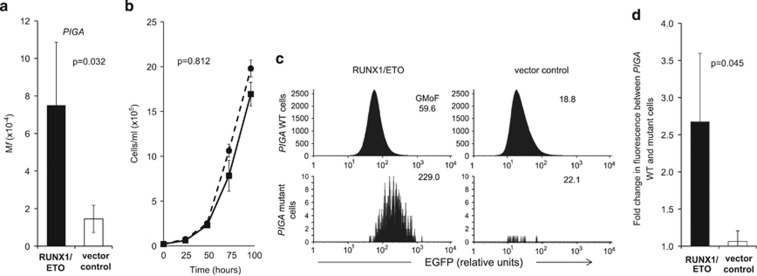
Expression of RUNX1/ETO increases spontaneous M*f*. (**a**) RUNX1/ETO clones (black bar) and vector control clones (white bar) were assayed for M*f* at the *PIGA* gene. RUNX1/ETO and vector control clones were cultured post cloning for 8–10 weeks before assessment of PIGA M*f*. M*f* was calculated by quantifying the number of PIGA-negative cells, which had passed all gating steps described in Supplementary Figure 2 and dividing this by the total number of cells that had passed all gating steps described in Supplementary Figure 2. M*f* values are the mean of five RUNX1/ETO clones and four vector control clones. RUNX1/ETO clones showed a significant increase (*P*=0.032) in *PIGA* M*f* compared with vector control clones. (**b**) Growth curves of RUNX1/ETO (solid line) and vector control cells (dashed line). Cells were seeded at 2 × 10^4^/ml and cell proliferation in six RUNX1/ETO clones and five vector control clones was measured every 24 h for 4 days. Mean cells/ml value of all clones is displayed and error bars represent the s.d. No significant difference was observed between the proliferation of RUNX1/ETO clones and vector control clones (*P*=0.812; ANOVA). (**c**) Example flow cytogram plots showing a higher EGFP level in *PIGA* mutant cells (bottom left panel) compared with WT PIGA cells (top left panel) from a single RUNX1/ETO clone. In contrast, differential EGFP expression between *PIGA* mutant and WT cells was not observed in vector control cells from a single clone (right panels) (further examples are shown in Supplementary Figure 4). Numbers represent EGFP geometric mean of fluorescence (GMoF). (**d**) EGFP levels in *PIGA* mutant and WT cells. EGFP levels (a surrogate for RUNX1/ETO) in *PIGA* mutant and WT cells were measured in RUNX1/ETO clones (black bars) and vector control clones (white bars) and represented as fold change of EGFP fluorescence between *PIGA* WT and mutant cells. Histogram shows the mean from five RUNX1/ETO clones and four vector control clones. Error bars represent the standard deviation. RUNX1/ETO *PIGA* mutants had significantly higher EGFP fluorescence than *PIGA* WT RUNX1/ETO cells from the same population, whereas no significant difference was observed between vector control *PIGA* mutant and WT cells (*P*=0.045; one tailed unpaired Student's *t*-test using fold change in fluorescence between PIGA WT and mutant cells and comparing RUNX1/ETO clones and vector control clones).

**Figure 2:**
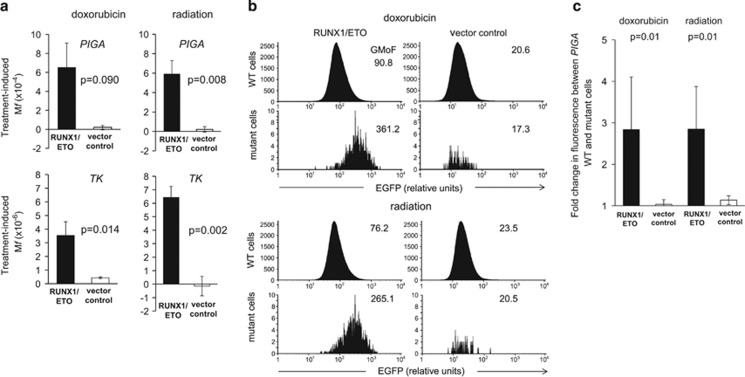
Expression of RUNX1/ETO increases M*f* after dosing with doxorubicin or ionising radiation. (**a**) RUNX1/ETO clones (black bars) and vector control clones (white bars) were cultured for 3 weeks post cloning before treating with doxorubicin, radiation or mock, with an additional 2 weeks for phenotype development before assaying for M*f* at the *PIGA* (top panels) and *TK* (bottom panels) genes. The frequency of mutations attributable to doxorubicin or radiation (treatment-induced M*f*) was calculated by subtracting the M*f* in mock-treated cells from the M*f* in doxorubicin or radiation-treated cells. Treatment-induced M*f* values displayed are the mean of five RUNX1/ETO clones and four vector control clones for *PIGA* and three RUNX1/ETO clones and four vector control clones for *TK*. RUNX1/ETO clones showed a significant increase in M*f* at *PIGA* (*P*=0.008; unpaired Student's *t*-test, two tailed) and *TK* (*P*=0.002) after radiation treatment. RUNX1/ETO clones showed a significant increase in M*f* at *TK* after doxorubicin treatment (*P*=0.014), and a non significant increase in M*f* at *PIGA* (*P*=0.09). (**b**) Example flow cytogram plots showing a higher EGFP level in *PIGA* mutant cells (bottom left panel for doxorubicin or radiation-treated cells) compared with WT *PIGA* cells (top left panels) from a single RUNX1/ETO clone. In contrast, differential EGFP expression between *PIGA* mutant and WT cells were not observed in vector control cells from a single clone after either doxorubicin or radiation treatment (right panels) (further examples are shown in Supplementary Figure 6). Numbers represent EGFP GMoF. (**c**) EGFP levels in *PIGA* mutant and WT cells after treatment with doxorubicin or ionising radiation. EGFP levels (a surrogate for RUNX1/ETO) in *PIGA* mutant and WT cells were measured in RUNX1/ETO clones (black bars) and vector control clones (white bars) and represented as fold change of EGFP fluorescence between *PIGA* WT and mutant cells. Histogram shows the mean from five RUNX1/ETO clones and four vector control clones. Error bars represent the standard deviation. RUNX1/ETO *PIGA* mutants had a significantly higher EGFP fluorescence than *PIGA* WT RUNX1/ETO cells from the same population after treatment with doxorubicin or radiation, whereas no significant difference was observed between vector control *PIGA* mutant and WT cells (*P*=0.01 for doxorubicin and *P*=0.01 for radiation; one tailed unpaired Student's *t*-test using fold change in fluorescence between PIGA WT and mutant cells and comparing RUNX1/ETO clones and vector control clones).
