# Supplementary material for: The leukemia-associated RUNX1/ETO oncoprotein confers a mutator phenotype
Source: Leukemia. 2015 Jun 30;30(1):251–4. doi: 10.1038/leu.2015.133 (PMC4705432; doi:10.1038/leu.2015.133)
Supplement: Supplementary Figure 1 [file leu2015133x1.pdf]

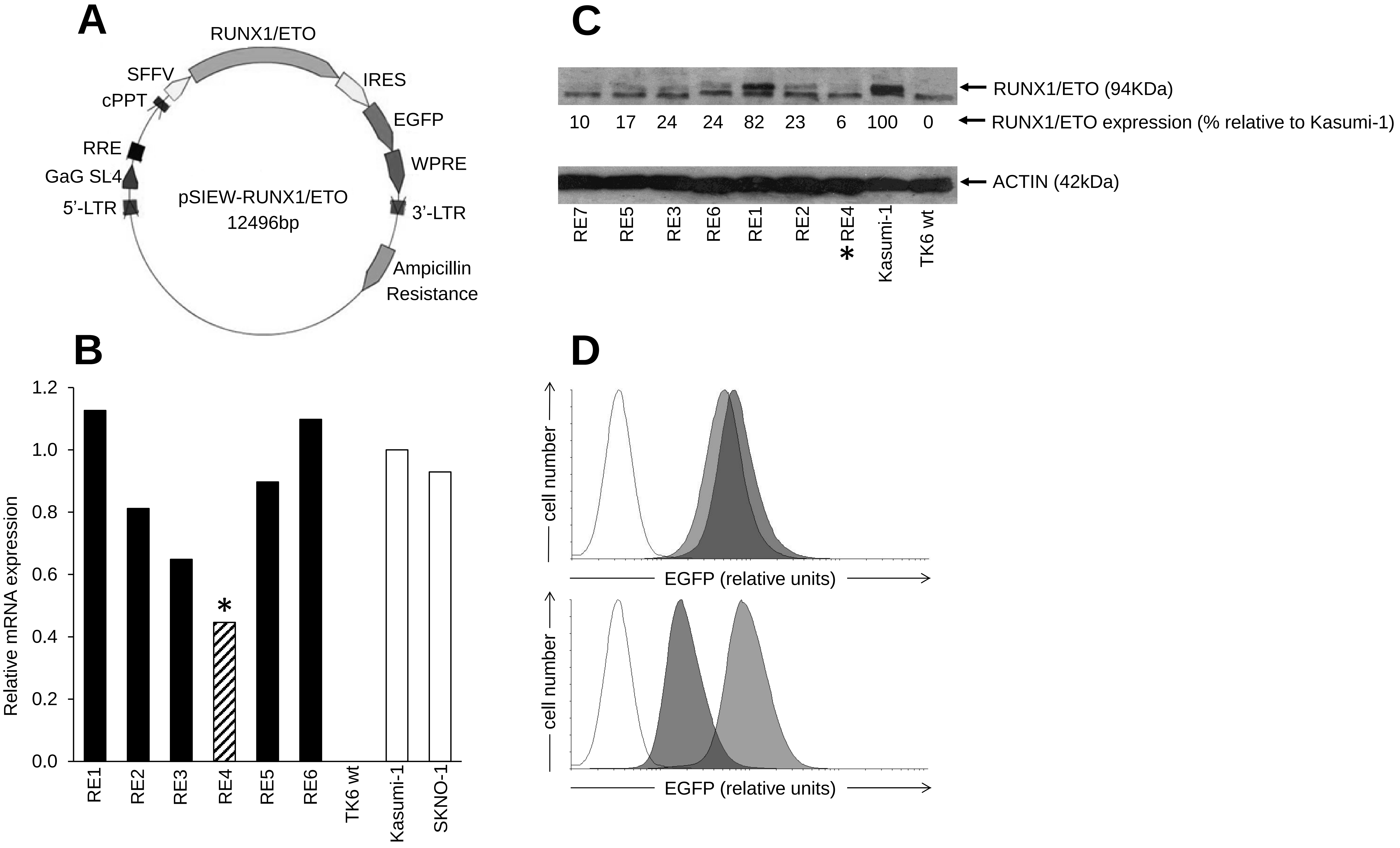

Supplementary Figure 1. Expression of RUNX1/ETO in TK6 clones.

(A) pSIEW-RUNX1/ETO vector map. CPPT, central polypurine tract; RRE, Rev response element; SFFV, Spleen focus forming virus; IRES, Internal ribosome entry site; WPRE, Woodchuck post-transcriptional regulatory element; GaG SL4, GaG Stem loop 4. (B) RUNX1/ETO (RE) TK6 clones (black bars), including a low-expressing clone (dashed bar with *) and the RUNX1/ETO^{+} patient-derived cell lines Kasumi-1 and SKNO-1 (white bars) were analysed for *RUNX1/ETO* transcript levels by real-time qPCR relative to that found in Kasumi-1 and normalised to levels of housekeeping gene *TBP*. Results displayed are the average of two independent experiments. (C) Western blot analysis of TK6 RUNX1/ETO clones for RUNX1/ETO protein and Actin. Lane denoted * is the low expressing RUNX1/ETO clone. RUNX1/ETO protein expression was determined using semi-quantitative densitometry on a Fuji LAS-3000 Luminescent Image Analyzer System, and is expressed as a percentage relative to Kasumi-1 following background subtraction (far right lane, parental TK6) and normalisation to actin. (D) Top panel: Example plots of RUNX1/ETO clones (grey plots) analysed for EGFP expression and compared to wild-type TK6 (white plot). Bottom panel: Example plots of vector control clones (grey plots) analysed for EGFP expression and compared to wild-type TK6 (white plot).
