# Supplementary material for: The leukemia-associated RUNX1/ETO oncoprotein confers a mutator phenotype
Source: Leukemia. 2015 Jun 30;30(1):251–4. doi: 10.1038/leu.2015.133 (PMC4705432; doi:10.1038/leu.2015.133)
Supplement: Supplementary Figure 2 [file leu2015133x2.pdf]

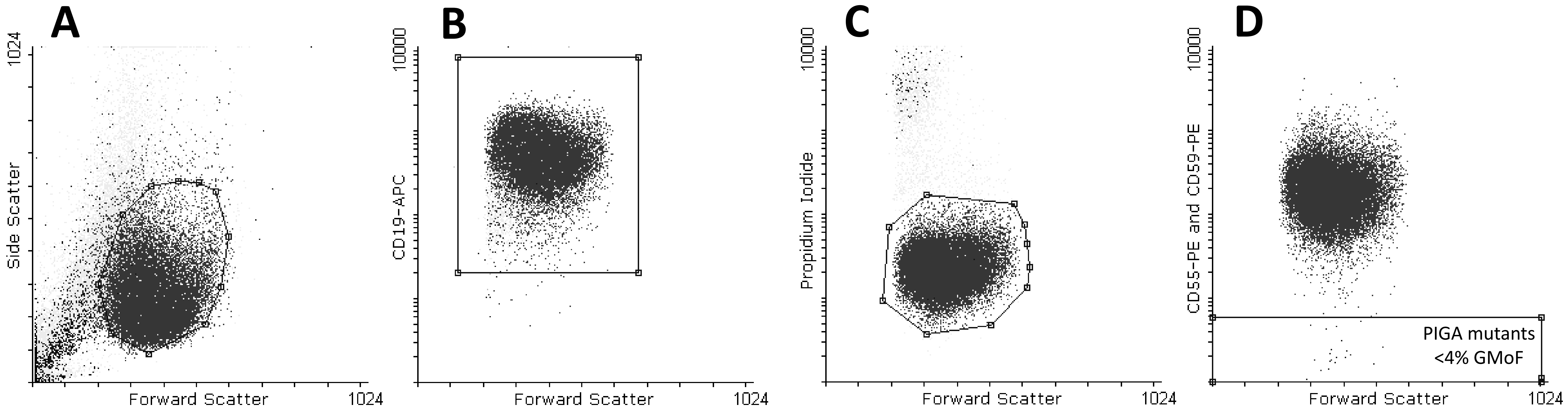

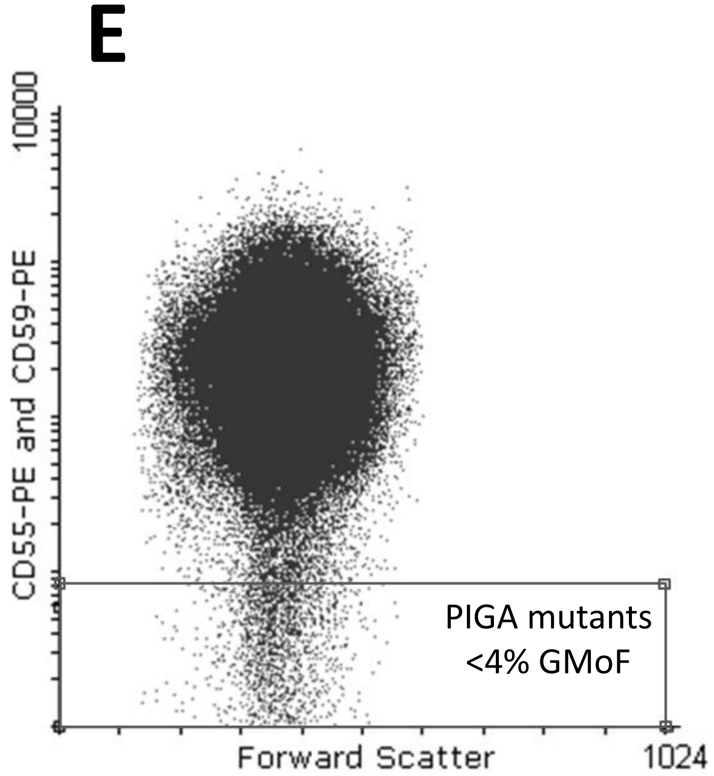

Supplementary Figure 2. Stages of gating involved in the *PIGA* mutation assay.

Cells analysed for *PIGA* mutation were gated by a number of steps to assure the detection of true *PIGA* mutants and minimal false-negative events. (A) Gating of cells based on Forward Scatter and Side Scatter to exclude cell clumps and cell debris. (B) Gating of cells with CD19-APC antibody, a B-cell surface marker as a positive control for successful antibody binding. (C) Gating of cells based on the exclusion of propidium iodide to exclude dead cells. (D) Gating of cells for CD55-PE and CD59-PE, PIGA-anchored cell surface proteins. The geometric mean of fluorescence in the FL-2 channel where CD55-PE and CD59-PE were detected (Y axis) was calculated and the threshold point for calculating PIGA mutants was defined as any events that had less than 4% of the geometric mean of the average of the whole population were considered to be *PIGA* mutants (boxed in panel D). (E) MT1 cells were used as a positive control for the PIGA assay. MT1 is a derivative of TK6 cells and have a significantly elevated mutation rate due to compound heterozygous mutations in the *MSH6* DNA mismatch repair gene. Cells were gated and *PIGA* mutant cells determined as described in panels A-D.
