# Supplementary material for: The leukemia-associated RUNX1/ETO oncoprotein confers a mutator phenotype
Source: Leukemia. 2015 Jun 30;30(1):251–4. doi: 10.1038/leu.2015.133 (PMC4705432; doi:10.1038/leu.2015.133)
Supplement: Supplementary Figure 3 [file leu2015133x3.pdf]

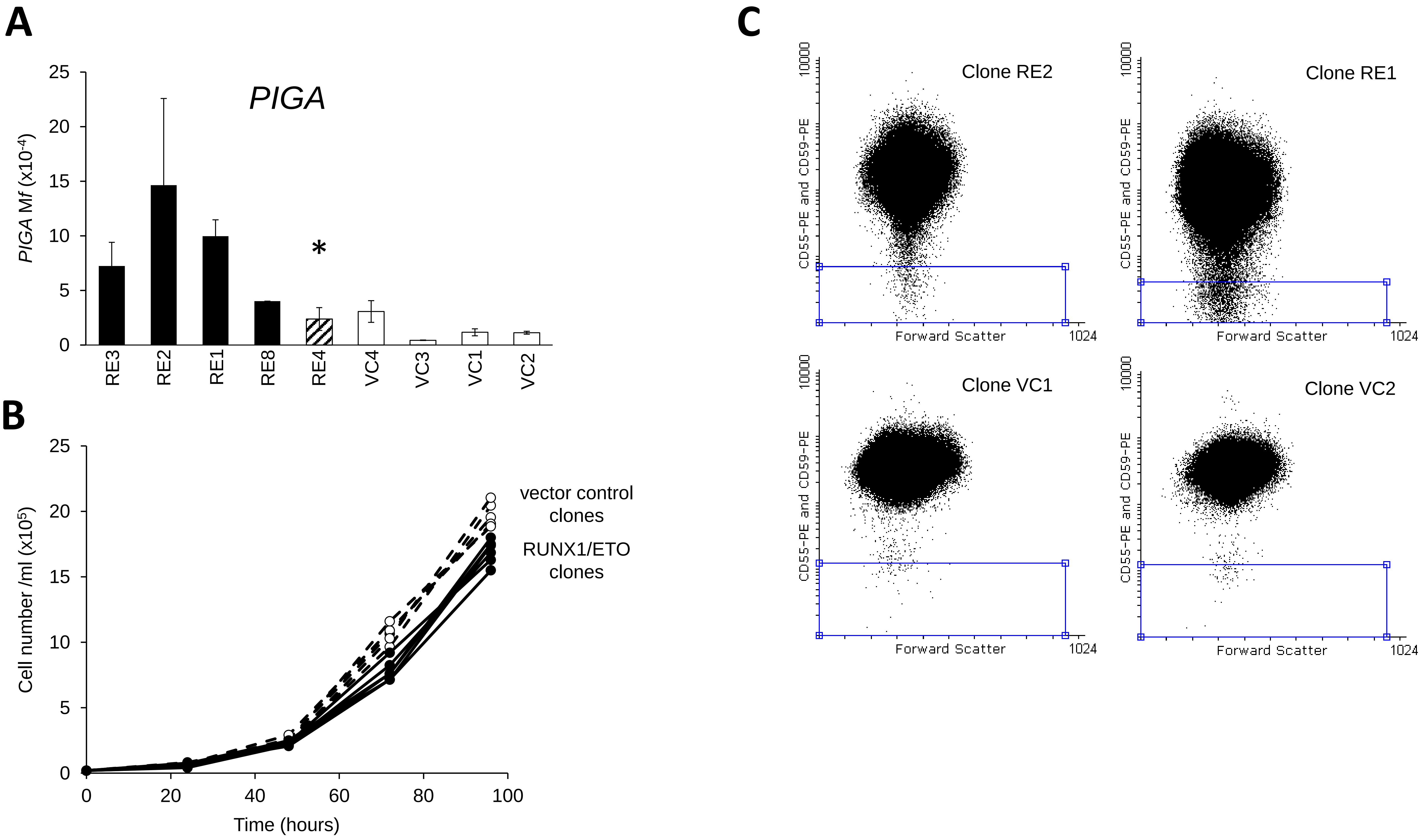

Supplementary Figure 3. Spontaneous *PIGA* mutation frequency and cell proliferation in individual RUNX1/ETO and vector control clones.

(A) RUNX1/ETO (RE) TK6 clones (black bars), including a very low-expressing clone (*), and vector control (VC) clones (white bars) were cultured post-cloning for 8-10 week prior to assessment of PIGA mutation frequency (M*f*). Results displayed are the average of three independent experiments and error bars represent the standard error of the mean (SEM) from 3 technical replicates in each experiment. Statistical analysis (excluding the low expressing clone) using Mann-Whitney U non-parametric test showed an increase in PIGA M*f* between RUNX1/ETO and vector control clones (p=0.032). (B) Growth curves of RUNX1/ETO cells and vector control cells. Cells were seeded at 2 x 10^4/ml and cell proliferation in 6 RUNX1/ETO clones (solid circles) and 5 vector control clones (open circles) was measured every 24 hours for 4 days. (C) Representative plots of CD55-PE and CD59-PE expression and gating for PIGA mutants in clones; TK6 RE3 (top left), TK6 RE1 (top right), TK6 VC1 (bottom left) and TK6 VC2 (bottom right). Events within blue box represent those scored as PIGA mutants.
