# Supplementary material for: The leukemia-associated RUNX1/ETO oncoprotein confers a mutator phenotype
Source: Leukemia. 2015 Jun 30;30(1):251–4. doi: 10.1038/leu.2015.133 (PMC4705432; doi:10.1038/leu.2015.133)
Supplement: Supplementary Figure 4 [file leu2015133x4.pdf]

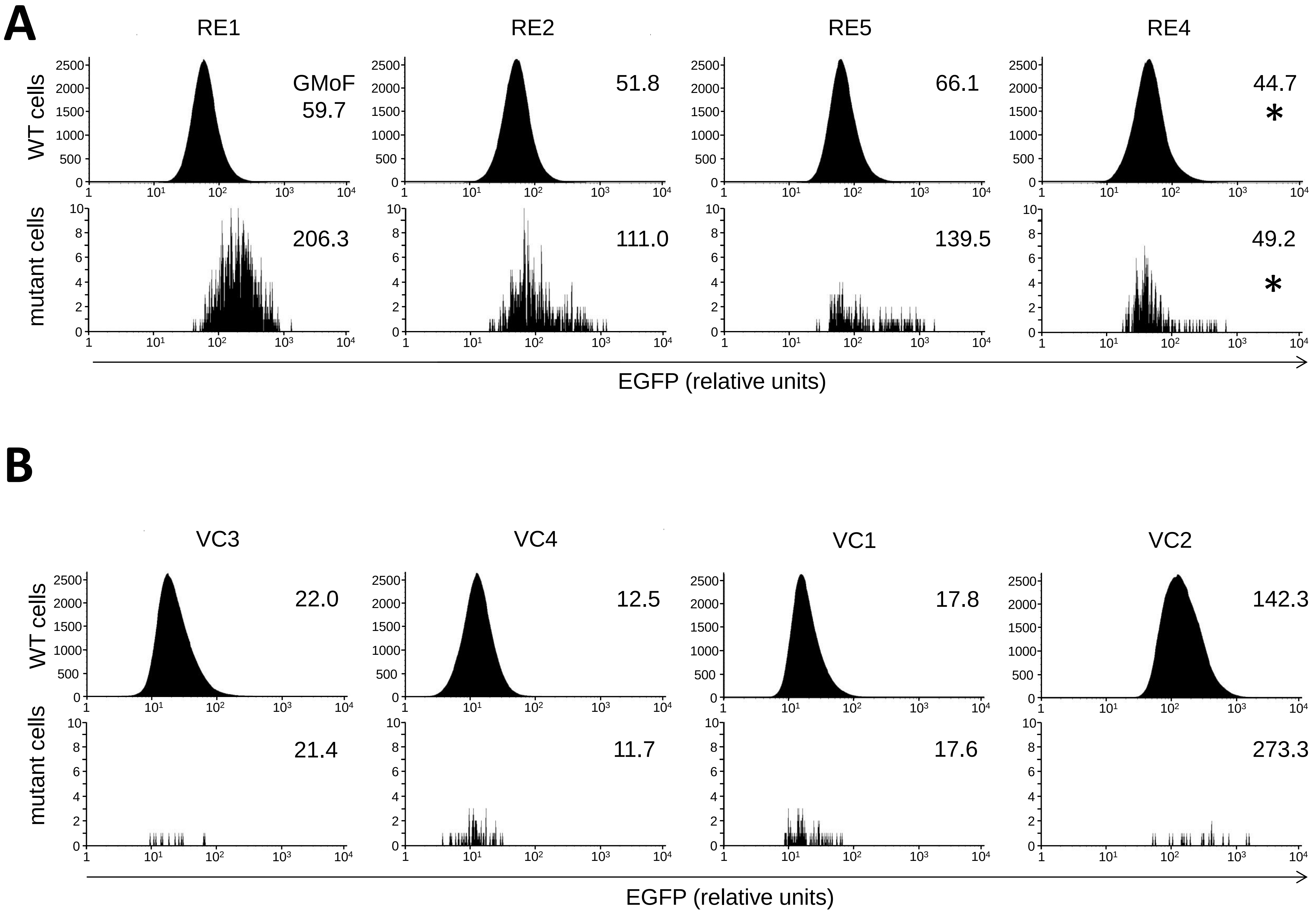

Supplementary Figure 4. *PIGA* mutant RUNX1/ETO clones have a significantly higher EGFP level than wild-type cells from the same population

(A) RUNX1/ETO TK6 clones, including a low-expressing clone (*) were analysed for spontaneous *PIGA* mutation after 8-10 weeks before assaying for mutation frequency (M*f*) at *PIGA*. Example flow cytogram plots show a higher EGFP geometric mean of fluorescence (GMoF) level in *PIGA* mutant cells (bottom panels) compared to wild-type *PIGA* cells (top panels) from individual RUNX1/ETO clones. The low RUNX1/ETO-expressing clone (*) showed no difference in EGFP level between *PIGA* wild-type and mutant cells. (B) Vector control TK6 clones were analysed for spontaneous *PIGA* mutation after 8-10 weeks before assaying for mutation frequency (M*f*) at *PIGA*. Example flow cytogram plots show similar EGFP geometric mean of fluorescence (GMoF) levels in *PIGA* mutant cells (bottom panels) compared to wild-type *PIGA* cells (top panels) from individual vector control clones. GMoF, geometric mean of fluorescence.
