# Supplementary material for: The leukemia-associated RUNX1/ETO oncoprotein confers a mutator phenotype
Source: Leukemia. 2015 Jun 30;30(1):251–4. doi: 10.1038/leu.2015.133 (PMC4705432; doi:10.1038/leu.2015.133)
Supplement: Supplementary Figure 5 [file leu2015133x5.pdf]

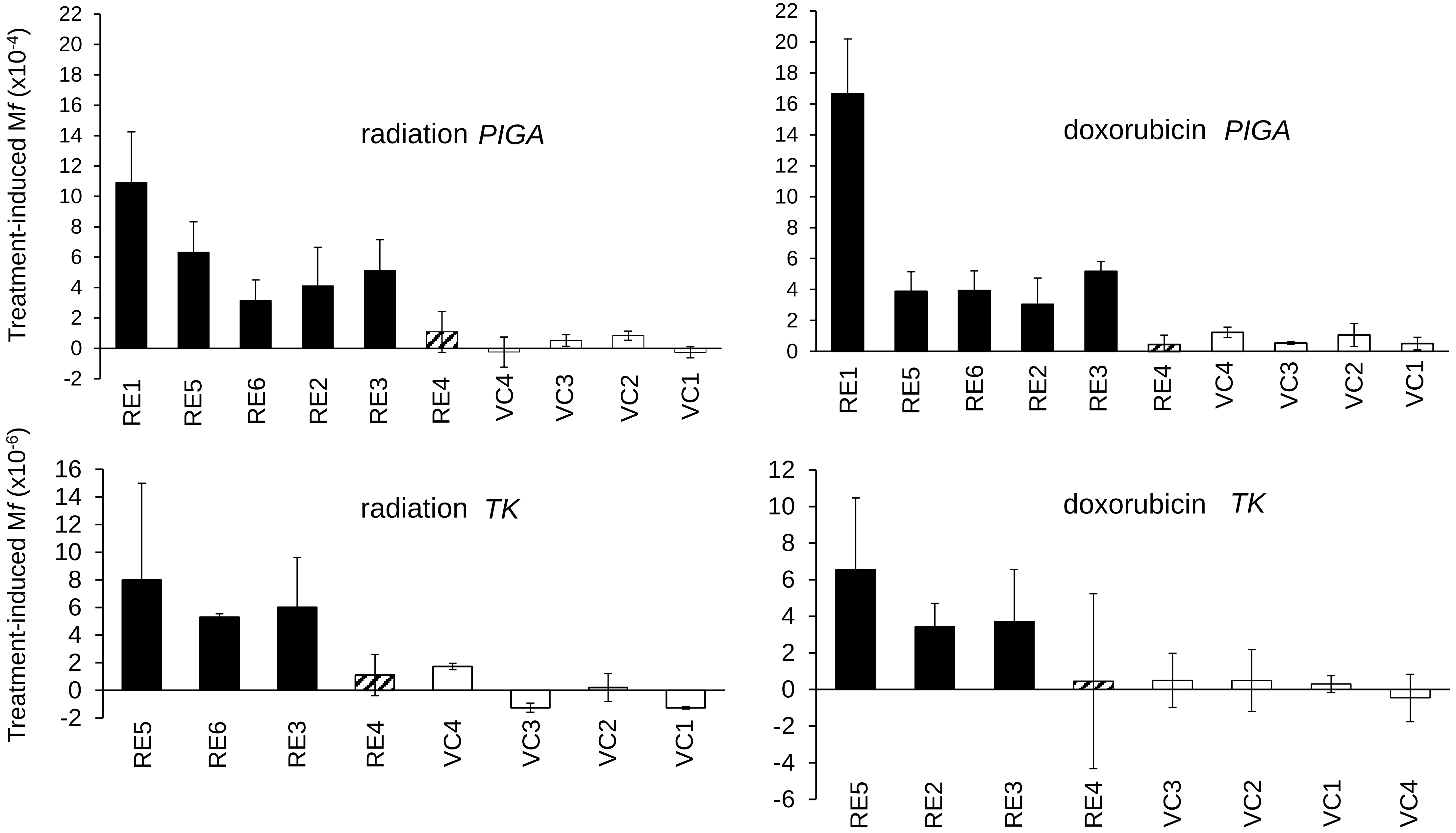


**Supplementary Figure 5.** Expression of RUNX1/ETO increases mutation frequency after dosing with doxorubicin or ionising radiation (individual clone data).

RUNX1/ETO clones (black bars), low expressing RUNX1/ETO clone (dashed bar) and vector control clones (white bars) were cultured for 3 weeks post cloning before treating with doxorubicin or radiation (as indicated at the top of each panel), allowing two weeks for phenotype development and then assaying for mutation frequency (M*f*) at the *PIGA* (top panels) and *TK* (bottom panels) genes. The frequency of mutations attributable to doxorubicin or radiation (treatment-induced M*f*) was calculated by subtracting the M*f* in mock-treated cells from the M*f* in doxorubicin or radiation-treated cells. All RUNX1/ETO clones tested had higher *PIGA* and *TK* M*f* than vector control clones after radiation or doxorubicin treatment, excepting the low expressing clone which did not exhibit any significant increase in *PIGA* M*f* over vector control clones. Negative values treatment-induced mutation frequency are due to some treated samples having slightly lower *PIGA* M*f* than non-treated controls.
