# Supplementary material for: The leukemia-associated RUNX1/ETO oncoprotein confers a mutator phenotype
Source: Leukemia. 2015 Jun 30;30(1):251–4. doi: 10.1038/leu.2015.133 (PMC4705432; doi:10.1038/leu.2015.133)
Supplement: Supplementary Figure 6 [file leu2015133x6.pdf]

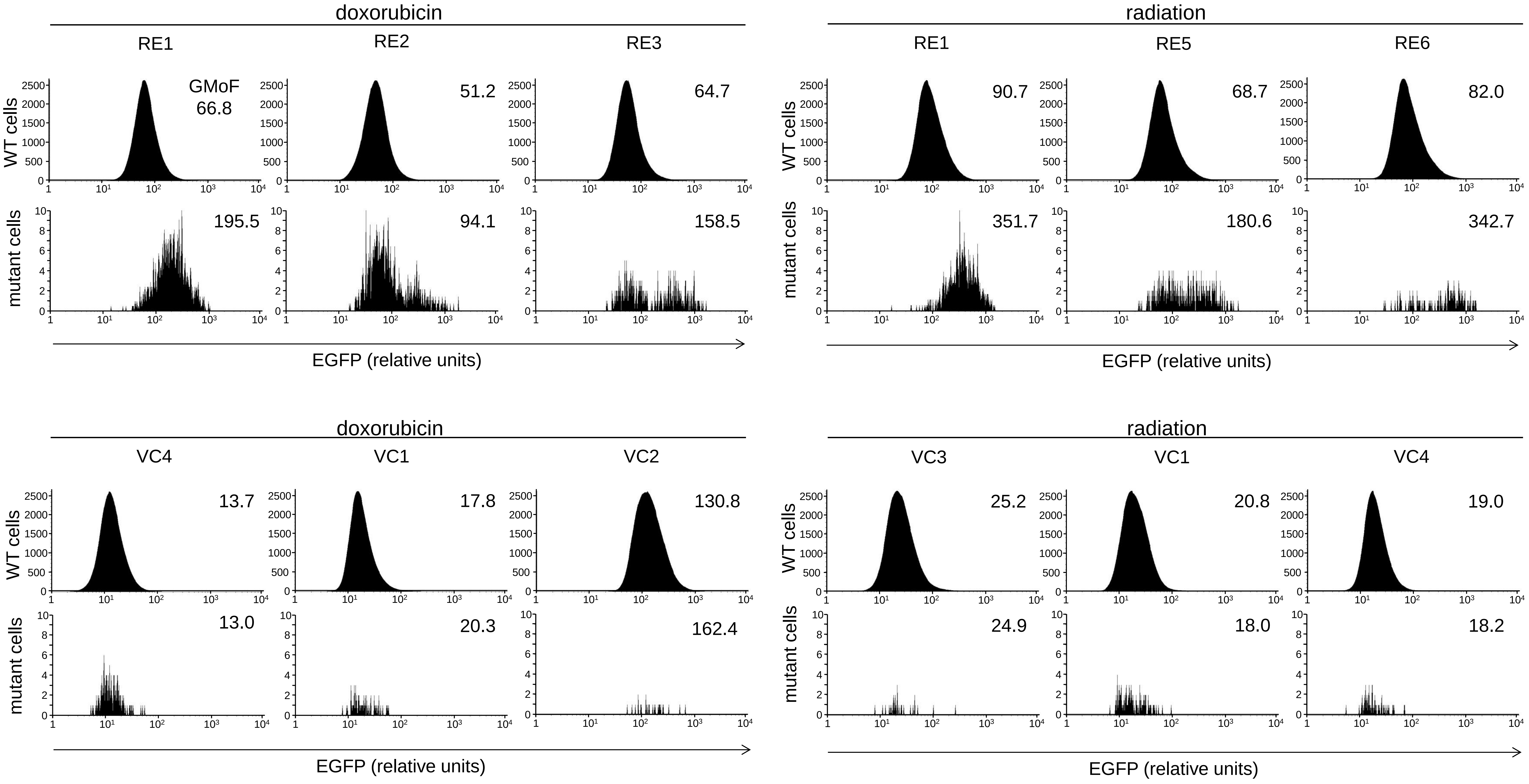

Supplementary Figure 6. *PIGA* mutant RUNX1/ETO clones have a significantly higher EGFP level than wild-type cells from the same population after doxorubicin or radiation treatment (representative plots).

RUNX1/ETO clones (top panel) and vector control clones (bottom panel) were cultured for 3 weeks post cloning before treating with doxorubicin (top left panel) or radiation (top right panel), allowing two weeks for phenotype development and then assaying for mutation frequency (M*f*) at the *PIGA* gene locus. Example flow cytogram plots are shown demonstrating a higher EGFP level in *PIGA* mutant cells (bottom panels) compared to wild-type PIGA cells (top panels) from individual RUNX1/ETO clones. No difference in EGFP levels between *PIGA* mutant and wild-type cells was observed in vector control clones after treatment with doxorubicin (bottom left panel) or radiation (bottom right panel). Numbers represent EGFP geometric mean of fluorescence (GMoF).
