# Supplementary material for: The leukemia-associated RUNX1/ETO oncoprotein confers a mutator phenotype
Source: Leukemia. 2015 Jun 30;30(1):251–4. doi: 10.1038/leu.2015.133 (PMC4705432; doi:10.1038/leu.2015.133)
Supplement: Supplementary Figure 7 [file leu2015133x7.pdf]

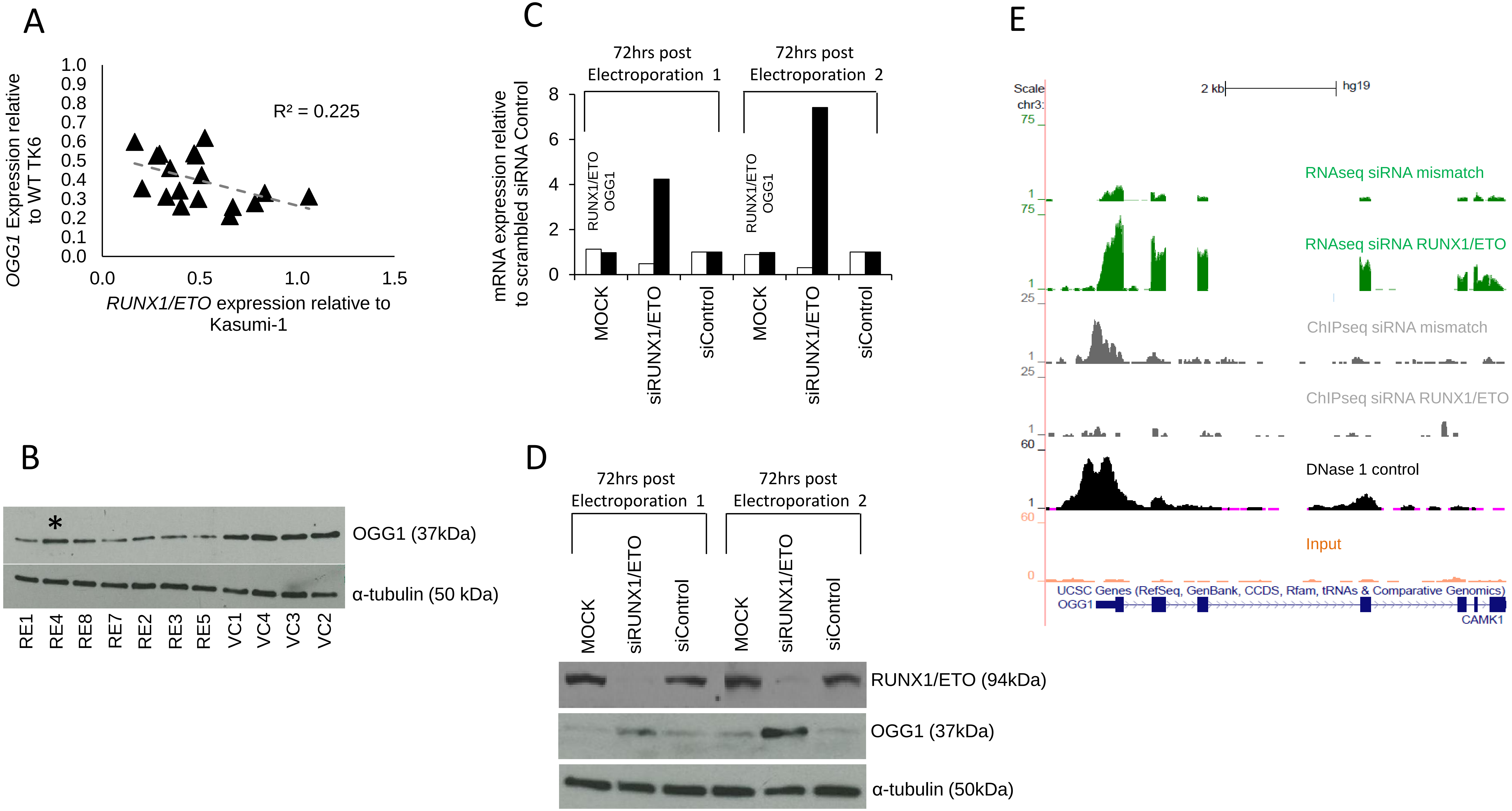


**Supplementary Figure 7.** RUNX1/ETO down regulates OGG1 via promoter binding, which can be rescued by treatment with siRNA targeted against RUNX1/ETO

(A) Expression of *RUNX1/ETO* and *OGG1* transcript was quantified in TK6 RUNX1/ETO clones by real-time PCR. *RUNX1/ETO* and *OGG1* transcript levels were inversely correlated ($R^2 = 0.225$). (B) TK6 RUNX1/ETO clones (RE) have reduced levels of OGG1 protein compared to vector control clones as measured by western blotting analysis. * indicates clone RE4, with low RUNX1/ETO transcript expression equivalent to 40% of that seen in Kasumi-1 (C) Knockdown of *RUNX1/ETO* by targeted siRNA was measured 72 hours post electroporation with 200nM of siRNA or no siRNA MOCK electroporated control. White bars represent *RUNX1/ETO* transcript levels and black bars represent *OGG1* transcript levels. *RUNX1/ETO* transcript was reduced by 52% and 69% and *OGG1* transcript increased by 4.2 and 7.4 fold, after the first and second electroporations, respectively. (D) Western blot analysis showing reduction in RUNX1/ETO protein and concurrent increase in OGG1 protein. (E) ChIPseq (grey lanes) and RNAseq (green lanes) analysis demonstrating that RUNX1/ETO binds to the promoter region of *OGG1* in Kasumi-1 cells. Colocalisation with a DNase hypersensitivity site indicates binding to open chromatin. Treatment with siRUNX1/ETO (siRE), but not with a mismatch control siRNA (siMM) leads to loss of RUNX1/ETO binding and increased *OGG1* transcript levels.
