# Supplementary material for: The leukemia-associated RUNX1/ETO oncoprotein confers a mutator phenotype
Source: Leukemia. 2015 Jun 30;30(1):251–4. doi: 10.1038/leu.2015.133 (PMC4705432; doi:10.1038/leu.2015.133)
Supplement: Supplementary Information [file leu2015133x8.pdf]

SUPPLEMENTARY MATERIALS AND METHODS

Lentiviral vectors and lentivirus production

The lentiviral construct pHR-cPPT-SIEW-RUNX1/ETO (p-SIEW-RR) was generated by inserting the *RUNX1/ETO* coding region into the BamHI site in the pHR-cPPT-SIEW lentiviral transfer vector (kindly provided by Michela Scherr, Hannover) thus linking RUNX1/ETO with enhanced green fluorescent protein (EGFP) expression via an internal ribosomal entry site (Supplementary Figure 1A). Lentivirus was produced as previously described (Bomken et al, 2013). Briefly, HEK293T cells were seeded in tissue culture plates and combined with calcium phosphate precipitates containing the lentiviral packaging plasmids pMD2.G, pCMVR8.91 and p-SIEW-RR. Lentivirus was collected 72 hours after initial transduction by ultracentrifugation. Target cells were transduced by spinfection at 1500g in 6-well tissue culture dishes for 2 hours at 32°C with 4μg/ml polybrene (Sigma Aldrich, Poole, UK). Cells were initially assessed for successful transduction by flow cytometry for EGFP and later real-time qRT-PCR and western blotting.

Cell cultures

TK6 (Kindly provided by Prof. W. Thilly, MIT, MA, USA) and Kasumi-1 were cultured in RPMI 1640 medium, supplemented with 10% FBS, 100 units/ml penicillin and 0.1mg/ml streptomycin. HEK293T lentiviral producer cells were cultured in DMEM (HEPES modification), supplemented with 10% FBS, 8nM L-glutamine and 1mM sodium pyruvate. SKNO-1 were cultured in RPMI 1640 medium supplemented with 20% FBS, 100 units/ml penicillin, 0.1mg/ml streptomycin and 10ng/ml granulocyte-macrophage colony stimulating factor. Kasumi-1, SKNO-1 and HEK293T cell lines were acquired from Leibniz Institut, Deutsche Sammlung von Mikroorganismen und Zellkulturen (DSMZ), Braunschweig, Germany. All cell culture media and supplements were acquired from Sigma Aldrich, unless otherwise stated.

Selection of successfully transduced cells

Three days after transduction with lentivirus, TK6 were transferred to 96-well plates at a mean density of 2 cells per well in 200µl of culture media and incubated for 10-14 days to isolate individual clonal populations. TK6 clones were assessed for successful lentiviral transduction initially by flow cytometry for EGFP and subsequently for RUNX1/ETO transcript and protein by real-time quantitative PCR and Western analysis, respectively.

Assessment of cell proliferation

Cells were seeded at 2 x 10^4 cells/ml and growth was assessed every 24 hours by cell counting using trypan blue exclusion with an improved Neubauer haemocytometer (VWR International Limited, Leicestershire, UK). A minimum of 2 separate preparations were counted for each cell sample and the average was taken to calculate cell concentration.

Real-time quantitative PCR

RNA was isolated using the RNeasy Mini Kit according to manufacturer's instructions (Qiagen Ltd, Crawley, UK) and 2µg was reverse transcribed to cDNA using the High Capacity cDNA Reverse Transcription Kit according to manufacturer's instructions (Applied Biosystems, Warrington, UK). Quantitative RT-PCR (qRT-PCR) was performed using SYBR Green Master Mix on a 7900HT Fast Real-Time PCR system (Applied Biosystems) and analysed with SDS version 2.3 software (Applied Biosystems). All primers for qRT-PCR applications were synthesised by Sigma Aldrich.

Western analysis

Whole cell protein extracts were generated from 10^6 cells using sodium dodecyl sulphate (SDS) extraction. Briefly, cell pellets were prepared by centrifugation at 300g before washing twice in PBS to remove protein contamination from media. Pellets were then resuspended in SDS sample buffer (62.5mM Tris-HCl pH 6.8, 2% (w/v) SDS, 20% (v/v) glycerol), followed by boiling samples at 100°C for 5 minutes. Samples were then centrifuged at 14,000g to pellet cell debris and protein-containing supernatants were stored at -80°C. Protein samples were

quantified using the Pierce® BCA Assay (Fisher Scientific UK Ltd, Leicestershire, UK) on a Spectromax®250 Microplate Spectrophotometer System (Molecular Devices Corporation, Crawley, UK). 20µg protein was electrophoresed on a Mini-PROTEAN® TGX™ 4-20% Tris-glycine gel (BioRad, Hemel Hempsted, UK) using a Mini PROTEAN Tetra system (BioRad) and transferred to PVDF membrane (BioRad). After hybridisation of antibodies, detection was performed with ECL plus Western Blotting Detection Kit (GE Healthcare, Amersham, UK). Primary antibodies used were goat anti-ETO (Santa Cruz, Heidelberg, Germany) and mouse anti-β-actin (Dako, Ely, UK). RUNX1/ETO protein expression was determined using semi-quantitative densitometry on a Fuji LAS-3000 Luminescent Image Analyzer System (model LAS-3000), and is expressed as a percentage relative to Kasumi-1 following background subtraction and normalisation to actin.

Irradiation of cells

Cell suspensions were irradiated in a 25cm^3 tissue culture flasks using a D3300 X-Ray system (Gulmay Medical Ltd, Surrey, UK) at a dose rate of 2.4Gy/min.

Cytotoxicity assays

Cells in logarithmic phase were irradiated or treated with drug (or vehicle control) and growth inhibition was calculated using trypan blue exclusion with an improved Neubauer haemocytometer (VWR International Limited) to count cells at multiple time points after exposure.

Electroporation of cell lines with siRNA

10^7 cells were pelleted by centrifugation at 300g for 5 minutes and resuspended in fresh media. The cell suspension was transferred to an electroporation cuvette with 4mm electrode gap (PEQLAB, Southampton, UK). The required amount of 20µM siRNA stock solution to give a final concentration of between 10-500nM was added to the cuvette. The

cuvette was electroporated at 330V (Kasumi-1) or 350V (SKNO-1) for 10ms using an EPI 2500 electroporator (Fischer, Heidelberg, Germany).

Determination of mutation frequency (Mf) at the thymidine kinase (TK) locus

Prior to experimental use, cell populations were purged of pre-existing TK mutants by 48-hour culture in CHAT medium (standard growth medium supplemented with 10μM 2-deoxycycline, 17.5μM thymidine, 200μM hypoxanthine and 0.2μM aminopterin; all chemicals were from Sigma Aldrich) followed by 72 hours in THC medium (as CHAT, but without aminopterin). For assessment of drug-induced Mf, exponentially growing cell populations were dosed by supplementing drug (or vehicle control) into standard growth medium. Following 4 hours exposure to doxorubicin at a final concentration of 100nM in 10ml cell culture medium in 25cm^3 flasks, cells were washed in PBS and M*f* was determined according to the method of Liber and Thilly (1982). The frequency of mutations attributable to doxorubicin or radiation (treatment-induced M*f*) was calculated by subtracting the M*f* in mock-treated cells from the M*f* in doxorubicin or radiation-treated cells.

Determination of surface CD55 and CD59 status by flow cytometry

Mutation using loss of CD55 and CD59 as a surrogate for loss of PIGA was performed as previously described (Chen et al, 2001; Krüger et al, 2014). Briefly, expression of two GPI-anchored proteins (CD55 and CD59) was determined on TK6 cells using PE conjugated antibodies. Cells were also assessed for expression of CD19 B-cell marker and exclusion of propidium iodide (PI) as a cell viability stain. CD55-PE (clone IA10), CD59-PE (clone p282 (H19)) and CD19-APC (clone SJ25C1) conjugated antibodies were all purchased from BD Biosciences (Oxford, UK). Base substitutions and small insertions/deletions in the *PIGA* gene are the predominant mechanism leading to loss of surface CD55/CD59 expression *in vivo* (diagnostic for paroxysmal nocturnal hemoglobinuria (Shen et al, 2014)) and cell line studies *in vitro* (Chen et al, 2001). However, we cannot exclude the possibility that other

mechanisms of *PIGA* inactivation (such as large gene deletions and gene silencing) or mutation in other genes may also lead to loss of CD55/CD59 expression.

PIGA^{-ve} cells were defined as those which were positive for CD19, negative for CD55 and CD59 and did not stain with PI. A four-colour FACSCalibur instrument (Becton Dickinson, Oxford, UK) with CellQuestPro software (Becton Dickinson) was used for analysis. PIGA M*f* was determined using the following equation: PIGA Mf = number of PIGA^{-ve} cells / total number of cells meeting the inclusion criteria (positive for CD19 and exclusion of propidium iodide).

ChIPseq, RNAseq and DNase 1 hypersensitivity site mapping

ChIPseq, RNAseq and Dnase1 hypersensitivity mapping were performed as previously described (Ptasinska et al, 2014).

References for Supplementary Materials and Methods

Bomken S, Buechler L, Rehe K, Ponthan F, Elder A, Blair H, et al. Lentiviral marking of patient-derived acute lymphoblastic leukaemic cells allows in vivo tracking of disease progression. *Leukemia* 2013; **27**: 718-721.

Chen R, Knez JJ, Merrick WC and Medof ME. Comparative efficiencies of C-terminal signals of native glycophosphatidylinositol (GPI)-anchored proproteins in conferring GPI-anchoring. *Journal of Cellular Biochemistry* 2001; **84**: 68-83.

Krüger CT, Hofmann M, Hartwig A. The *in vitro* PIG-A gene mutation assay: mutagenicity testing via flow cytometry based on the glycosylphosphatidylinositol (GPI) status of TK6 cells. *Arch Toxicology*, 2015, *in press*.

Liber HL and Thilly WG. Mutation assay at the thymidine kinase locus in diploid human lymphoblasts. *Mutation Research* 1982; **94**:467-485.

Ptasinska A, Assi SA, Martinez-Soria N, Imperato MR, Piper J, Cauchy P,et al. Identification of a dynamic core transcriptional network in t(8;21) AML that regulates differentiation block and self-renewal. *Cell Reports* 2014; **8**: 1974-1988.

Shen W, Clemente MJ, Hosono N, Yoshida K, Przychodzen B, Yoshizato T, Shiraishi Y, Miyano S, Ogawa S, Maciejewski JP, Makishima H. Deep sequencing reveals stepwise mutation acquisition in paroxysmal nocturnal hemoglobinuria. *Journal of Clinical Investigation* 2014, **124**: 4529-4538.
