# Supplementary material for: The leukemia-associated RUNX1/ETO oncoprotein confers a mutator phenotype
Source: Leukemia. 2015 Jun 30;30(1):251–4. doi: 10.1038/leu.2015.133 (PMC4705432; doi:10.1038/leu.2015.133)
Supplement: Supplementary Table 1 [file leu2015133x9.pdf]

Cell Clone	Treatment	EGFP geometric mean of fluorescence of all cells meeting inclusion criteria	EGFP geometric mean of fluorescence of *PIGA* mutants	Number of events meeting inclusion criteria	Number of *PIGA* mutant events
RUNX1/ETO 1	None	69.1	176.2	361601	424
	Radiation	64.3	187.6	417341	1129
	Doxorubicin	66.3	142.4	302062	884
RUNX1/ETO 2	None	51.5	106.3	431310	314
	Radiation	46.9	90.1	575566	615
	Doxorubicin	51.0	96.1	539616	481
RUNX1/ETO 3	None	58.8	89.7	349401	200
	Radiation	50.7	81.2	640728	294
	Doxorubicin	59.8	149.7	496331	259
RUNX1/ETO 4*	None	47.6	53.0	304650	59
	Radiation	44.4	49.4	324003	82
	Doxorubicin	48.9	71.9	471229	79
RUNX1/ETO 5	None	69.7	154.7	424353	59
	Radiation	57.4	136.0	348358	279
	Doxorubicin	79.9	317.4	402673	151
RUNX1/ETO 6	None	60.2	233.2	426970	59
	Radiation	62.9	356.4	427604	544
	Doxorubicin	57.7	347.9	472004	284
Vector backbone 1	None	13.3	11.8	376729	37
	Radiation	17.7	19.4	311780	89
	Doxorubicin	18.9	18.5	398111	110
Vector backbone 2	None	115.2	121.5	393168	19
	Radiation	131.5	134.2	490087	48
	Doxorubicin	102.1	111.9	463218	67
Vector backbone 3	None	19.7	16.4	407868	20
	Radiation	21.2	21.9	493836	68
	Doxorubicin	18.3	17.0	406211	49
Vector backbone 4	None	13.1	11.8	286568	108
	Radiation	13.6	12.8	404376	184
	Doxorubicin	14.3	11.4	435397	236

Supplementary table 1. Representative data showing EGFP geometric mean of fluorescence (GMoF) of all cells meeting the inclusion criteria and *PIGA* mutant cells within that population.

Data shown is from a representative experiment, measuring spontaneous, doxorubicin-induced and radiation-induced *PIGA* mutants. Displayed is the EGFP geometric mean of fluorescence of all cells meeting inclusion criteria and *PIGA* mutant cells (outlined in supplementary Figure 2). RUNX1/ETO 4* is the low-expressing RUNX1/ETO clone highlighted similarly throughout figures.
