# Supplementary material for: The leukemia-associated RUNX1/ETO oncoprotein confers a mutator phenotype
Source: Leukemia. 2015 Jun 30;30(1):251–4. doi: 10.1038/leu.2015.133 (PMC4705432; doi:10.1038/leu.2015.133)
Supplement: Supplementary Table 2 [file leu2015133x10.pdf]

Base substitution	Normal	t(8;21)	t(15;17)	t(9;22)	inv(16)	MLL translocation	Complex Karyotype	Other (Intermediate Risk)	Other (Poor Risk)	All cases	All cases (excluding t(8;21))
A:T>G:C Transition	2619	10	937	8	9	5	52	39	15	3694	3684
G:C>A:T Transition	7736	55	2486	14	54	19	234	197	83	10878	10823
A:T>C:G Transversion	716	3	282	1	2	1	13	13	5	1036	1033
A:T>T:A Transversion	1182	2	482	2	2	2	24	14	6	1716	1714
G:C>T:A Transversion	1841	15	644	1	11	2	40	32	14	2600	2585
G:C>C:G Transversion	992	8	402	1	6	2	32	13	6	1462	1454
Total	15086	93	5233	27	84	31	395	308	129	21386	21293
Number of cases	92	7	18	3	11	7	24	22	9	193	186
% G:C>T:A transversions	12.20	16.13	12.31	3.70	13.10	6.45	10.13	10.39	10.85	12.16	12.14

Supplementary table 2. Somatic base substitutions in 193 AML cases at disease presentation.

Data was available from 193 AML cases, which included information on 21386 somatic base substitution mutations from 193 AML cases at disease presentation. Whole genome sequencing data was available from 50 cases (38 cases with a normal karyotype and 12 with t(15;17)) and exome sequencing data was available from 137 cases (54 normal karyotype, 7 t(8;21), 6 t(15;17), 3 t(9;22), 11 inv(16), 7 MLL translocation, 24 complex karyotype, 22 other cases of intermediate risk and 9 other cases of poor risk), as described by The Cancer Genome Atlas Research Network (2013) Genomic and epigenomic landscapes of adult de novo acute myeloid leukemia. N Engl J Med. 368:2059-2074.
